# Live births after polar body biopsy and frozen-thawed cleavage stage embryo transfer: case report

**DOI:** 10.5935/1518-0557.20160049

**Published:** 2016

**Authors:** Fernando Guimarães, Matheus Roque, Marcello Valle, Alessandra Kostolias, Rodrigo A de Azevedo, Ciro D Martinhago, Marcos Sampaio, Selmo Geber

**Affiliations:** 1 ORIGEN - Center for Reproductive Medicine, Rio de Janeiro/RJ - Brazil; 2 UFMG - Universidade Federal de Minas Gerais, Belo Horizonte/MG - Brazil; 3 Chromosome Genomic Medicine, São Paulo/SP - Brazil; 4 ORIGEN - Center for Reproductive Medicine, Belo Horizonte/MG - Brazil

**Keywords:** Polar body biopsy, pre-implantation genetic screening, pre-implantation genetic diagnosis, PGS, PGD

## Abstract

Pre-implantation genetic diagnosis (PGD) or screening (PGS) technology, has emerged and developed in the past few years, benefiting couples as it allows the selection and transfer of healthy embryos during IVF treatments. These techniques can be performed in oocytes (polar-body biopsy) or embryos (blastomere or trophectoderm biopsy). In this case report, we describe the first two live births to be published in Brazil after a polar-body (PB) biopsy. In case 1, a 42-year-old was submitted to PB biopsy with PGS due to advanced maternal age and poor ovarian reserve. Five MII oocytes underwent first and second polar body biopsy and four cleavage embryos were cryopreserved. The PGS analysis resulted in two euploid embryos (next generation sequence). A frozen-thawed embryo transfer (FET) was performed after endometrial priming and a healthy baby was delivered after a cesarean section (37 weeks, female, 3390g, 47.5 cm). In case 2, a 40-year old patient with balanced translocation and poor ovarian response was submitted to PB biopsy. Two MII oocytes underwent first and second polar body biopsy and two embryos were cryopreserved in cleavage stage. The analysis resulted in one euploid embryo that was transferred after endometrial priming. A preterm healthy baby (34 weeks, female, 2100g, 40 cm) was delivered via cesarean section. In conclusion, although the blastocyst biopsy is the norm when performing PGS/PGD during IVF treatments, other alternatives (as PB biopsy) should be considered in some specific situations.

## INTRODUCTION

In recent years, the development of assisted reproduction techniques (ART) has provided enormous advances to the treatment of infertile couples. One of the areas of development has been pre-implantation genetic analysis, with the use of preimplantation genetic diagnosis (PGD) or screening (PGS) technology. These techniques have benefited healthy couples and also those with a history of genetic disorders, enabling the selection and transfer of healthy embryos (Harper, 2009).

Chromosome aneuploidy is one of the major concerns related to advanced maternal age in women seeking pregnancy. It is associated with pregnancy failure, miscarriage and chromosomal anomalies in the offspring after natural or assisted conceptions (Christopikou *et al*., 2013). Pre-implantation genetic evaluation is a powerful tool to achieve a healthy pregnancy, avoiding potential adverse events related to advanced maternal age and chromosomal anomalies (Cimadomo *et al*., 2016).

Pre-implantation analysis can be performed in three developmental stages: oocyte (polar body biopsy); cleavage stage (blastomere biopsy); and finally: blastocyst stage (trophectoderm biopsy) (Kanavakis & Traeger-Synodinos, 2002). To perform the biopsy, it is important to cause a disruption of the zona pellucida of the oocyte or embryo occurs, which can be performed mechanically, chemically or using laser (Brezina *et al*., 2012). The key point of PGD/PGS is to have access to the genetic material to be evaluated, without compromising the material analyzed and the quality of the oocyte/embryo (Xu & Montag, 2012).

Polar body (PB) biopsy was introduced in 1990 (Verlinsky *et al*., 1990), and it is associated with a less invasive technique, presenting advantages, because it maintains embryo integrity, as only meiotic products are used to assess oocyte conditions. Removal of the first and/or second polar body is an indirect approach to evaluate oocyte genetic or chromosomal status (Montag *et al*., 2013). Moreover, the use of polar body biopsy is an option for countries with strict regulations regarding embryo biopsy, as it is the case in Germany and Switzerland (Harton *et al*., 2011; Xu & Montag, 2012). A pilot study (ESHRE PGS Task force) for PGS in women of advanced maternal age was conducted. Polar bodies following intracytoplasmic sperm injection (ICSI) were evaluated by CGH array to detect copy number changes and to predict aneuploidy status in the corresponding embryos. This study demonstrated that polar body and CGH array analysis were efficient and was highly concordant (94%) with the statuses of the corresponding zygotes (Christopikou *et al*., 2013). A recent publication from 2011 reviewed 938 cycles of PGD for 146 types of Mendelian monogenic diseases. They performed 9036 PB biopsies which were evaluated by multiplex PCR, with 1578 healthy embryos, which were later transferred. That resulted in 329 pregnancies and 345 births. This data, demonstrated an important conclusion regarding the safety and efficacy of the use of PGD with PB biopsy (Kuliev & Rechitsky, 2011). Nevertheless, it is hampered by the impossibility of diagnosing paternally derived defects, and those originating after fertilization or first cleavage events (Gianarolli L, 2000).

The main objective of this study was to describe the live birth of two healthy babies in Brazil, after performing a PB biopsy.

## CASE DESCRIPTION

### Case 1

A 42-year-old patient was referred to our center in November 2014 for an infertility workup. The patient provided a signed informed consent. The couple has a 7 -year-old son with autism. There was no male factor associated. She had a regular menstrual cycle and altered basal hormonal levels, as measured on day 2: follicle-stimulating hormone (FSH), 16 mIU/mL; luteinizing hormone (LH), 6,1 IU/mL; E2, 65 pg/mL. The antral follicle count (AFC) performed on day 3 showed 6 antral follicles.

Ovulation induction started on day 2 of her menstrual cycle in a step-down GnRH antagonist protocol, with 300 IU of recombinant FSH (rFSH; Gonal-F; Merck, Darmstadt, Germany) and 150 IU rFSH with 75 IU rLH (Pergoveris Merck SA, Aubonne, Swiss). The GnRH antagonist (Cetrotide; Merck) was introduced from day 5 of stimulation. Final oocyte maturation was induced when three follicles reached 18 mm in diameter, after 10 days of stimulation, with a bolus of 0.2 mg of GnRHa (triptorelin; Gonapeptyl daily; Ferring; Kiel; Germany). The ultrasound examination on the trigger day showed three follicles ≥ 18 mm, and three follicles between 14-18 mm in diameter. Oocyte retrieval was performed 36 hours after the trigger. A total of five oocytes were retrieved; five were metaphase II (MII), and all of them were inseminated (day 0) by intracytoplasmic sperm injection (ICSI). The first polar body (PB) biopsy was performed at the moment of the ICSI procedure. On the following day of ICSI (day 1), five oocytes were normally fertilized and all oocytes had the second polar body removed. On day 3, there were four cleaved embryos, and both the first and second polar bodies of these embryos were sent for analysis by NGS (next generation sequencing). Fifteen days after, we received the report showing two euploid embryos and two aneuploidy embryos.

**Figure 1:**
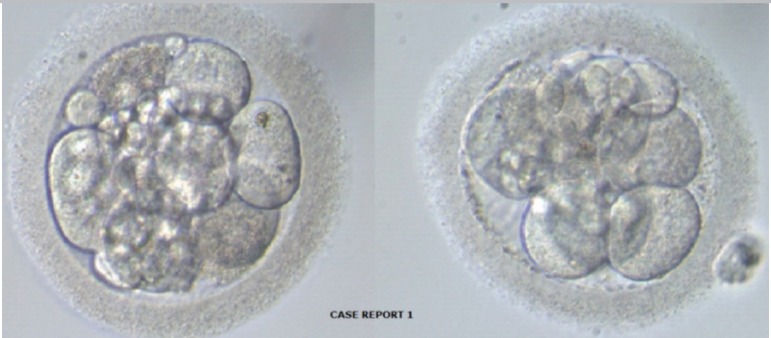
Two euploid embryos transferred - patient 1

Four months later, the patient started endometrial priming with estradiol valerate (6mg/day) on the second day of the cycle. On the 12th day of priming, she had normal hormonal measurements and an ultrasound scan showed an endometrial thickness of 7.5mm. The patient was started on vaginal progesterone. On the fourth day of progesterone administration, two euploid embryos (Fig. 1) were thawed and transferred on the same day. The patient became pregnant. In the 37th week of pregnancy, after a cesarean section, a healthy baby (female, 3390g; 47.5cm) was delivered. There were no obstetrical / perinatal complications.

### Case 2

A 40-year-old patient with balanced translocation was referred to our center. The patient provided a signed informed consent. There was no male factor associated. She had a regular menstrual cycle and normal basal hormonal levels, as measured on day 2: follicle-stimulating hormone (FSH) of 11,2 mIU/mL; luteinizing hormone (LH) of 5,6 IU/mL, and E2 of 49 pg/mL. The basal ultrasound showed four antral follicles. The karyotype showed a balanced translocation - 45XX rob(13.14)q10. Her partner had a normal karyotype.

**Figure 2:**
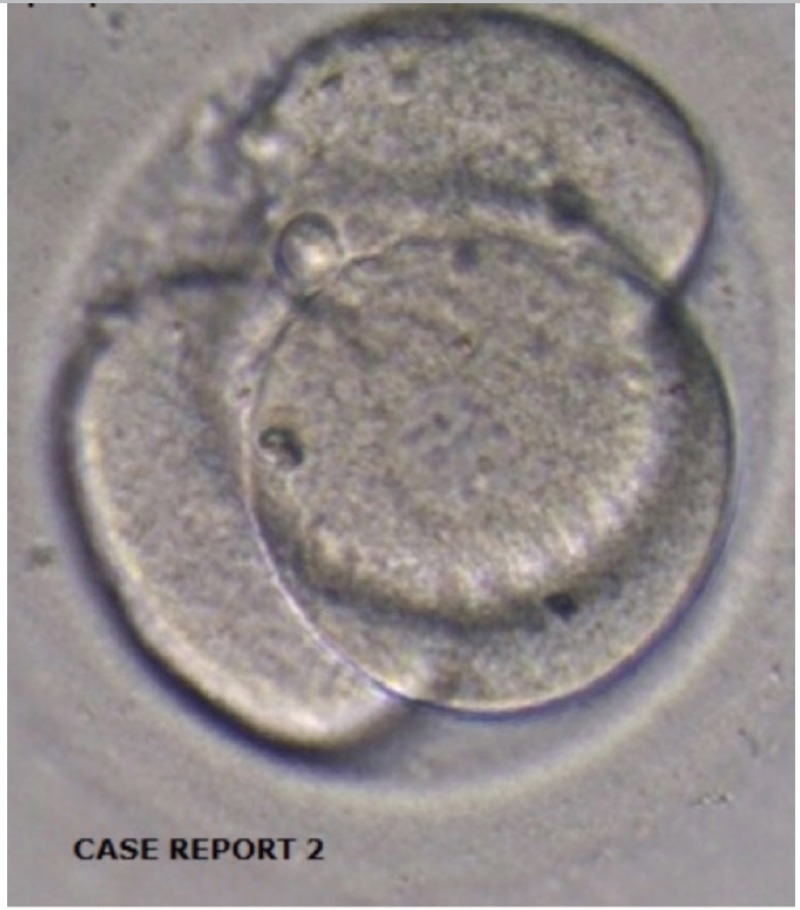
One euploid embryo - patient 2

Ovulation induction started on day 2 of her menstrual cycle in a step-down GnRH antagonist protocol, with 300 IU of recombinant FSH (rFSH; Gonal-F; Merck, Darmstadt, Germany) and 150 recombinant FSH with recombinant LH (Pergoveris Merck SA, Aubonne, Swiss). The GnRH antagonist (Cetrotide; Merck) was introduced as of day 5 of stimulation. Final oocyte maturation was induced when three follicles reached 18 mm in diameter, after 10 days of stimulation, with a bolus of 0.2 mg of GnRHa (triptorelin; Gonapeptyl daily; Ferring; Kiel; Germany). The ultrasound scan showed three follicles ≥ 18 mm in diameter, and two follicles between 16-18 mm in diameter. Oocyte retrieval was performed 36 hours after the trigger. A total of three oocytes were retrieved and two of them were MII. The mature oocytes were inseminated (day 0) by intracytoplasmic sperm injection (ICSI). The first polar body (PB) biopsy was performed at the moment of the ICSI procedure. On the following day of ICSI (day 1), two oocytes were normally fertilized and all oocytes had the second polar body removed. On day 2, there were two cleaved embryos, and both the first and second polar bodies of these embryos were sent for analysis by NGS (next generation sequencing). The NGS analysis showed one euploid embryo and one aneuploid embryo. Two months later, she started endometrial priming with estradiol valerate administration on the second day of the cycle, 6 mg orally daily. On the 12th day of priming she had normal hormonal measurements and an ultrasound scan showed an endometrial thickness of 8mm. Micronized vaginal progesterone (Crinone) was introduced, and on the third day of administration, one euploid embryo (Fig. 2) was thawed and transferred on the same day. The patient became pregnant and delivered a pre-term baby on the 34th week of gestation (female, 2100g; 40cm).

## DISCUSSION

To our knowledge these are the first two live births to be published after PB biopsy in Brazil. The diagnosis of polar body refers to an indirect method of genetically evaluating the oocyte and it seems to be a feasible technique to be employed in some specific situations. This paper presents two patients in which three recent, developing, and highly promising technologies were employed successfully: PB biopsy; cytogenetic evaluation with NGS; and embryo vitrification.

Currently, specific indications for performing PGD/PGS are: patients with advanced maternal age, monogenic diseases, maternal translocations, recurrent implantation failure and recurrent spontaneous abortion (Wang *et al*., 2010). Although the blastocyst biopsy is the current norm in clinical practice for PGS/PGD, PB biopsy may be an alternative for selected cases. However it is very important that there is an accurate alignment between the clinical evaluation of the patient with the desired type of laboratory technique, as well as proficiency and expertise (Van der Ven *et al*, 2008).

There are important positive considerations concerning the PB biopsy. First, the occurrence in some countries of stringent regulations or restrictions on the use of biopsy in embryonic stages as Cleavage and Blastocyst, enable only the performance of PB biopsy. Second, this type of procedure is characterized by reduced invasiveness and may not be as harmful as biopsies in more advanced stages, such as Cleavage and Blastocyst (Harton *et al*; 2011). Third, it may be considered in patients with poor ovarian response, with indication of PGD/PGS. Due to the low number of oocytes in this group of patients, sometimes there are no available embryos to be evaluated in the blastocyst stage. The two reported cases in this manuscript were related to poor ovarian response. All the available strategies were discussed with the patients and the PB biopsy was selected.

It is important to consider some issues and limitations of this technique. The predictive value of aneuploidy is considered impaired when compared blastomere or trophectoderm biopsies, which often require confirmatory analysis. Furthermore, this technique cannot detect genetic errors from the paternal side. Also, when there is a large number of oocytes to be tested, there is a decreased cost-effectiveness of this technique. Another issue that may arise is that polar bodies are very small in size, and eventually the biopsy samples may degenerate or simply not yield results (Xu & Montag, 2012).

Therefore, if PB biopsy will be performed, safe removal of the two polar bodies is mandatory. Thus, both can be analyzed by specific techniques for genetic analysis and a healthy embryo can be selected for embryo transfer (Wei *et al*., 2015). Important considerations must be taken into account when using this strategy: laboratory techniques, biopsy procedure, the freezing and thawing process, and finally the genetic analysis. Critical steps in this procedure are the opening of the zona pellucida of the oocyte, and the correct and non-traumatic removal of the first and second polar bodies (Magli *et al*., 2004).

As many embryo transfers are not performed in a fresh cycle when PGD/PGS is carried out, appropriate and validated embryo cryopreservation protocols are essential for achieving good results in this situation, to improve survival and implantation rates (Kuwayama M., 2007; Lee & Munne, 2000). In this study, all euploid embryos survived after the thawing process. In our IVF center, we have achieved good outcomes with embryo cryopreservation with over 90% survival rates when performing vitrification in the cleavage stage (Roque *et al*., 2015). Moreover, a well-trained staff with experience in embryo biopsy techniques is of great importance to reduce misdiagnoses. The introduction of new technologies in genetic analysis (such as aCGH, SNP microarrays, qPCR and NGS) was paramount in order to achieve the expected clinical benefits that could not be demonstrated by previous techniques such as the FISH analysis. These new methods enable the simultaneous evaluation and identification of embryos with specific chromosomal abnormalities (unbalanced translocations for example) and aneuploidy, thus enabling the selection of euploid embryo for later transfer. However, there is a need for more randomized controlled trials evaluating these new technologies, so as to calculate the real benefits of these strategies, and to pinpoint in which groups of patients it should be performed (Martin *et al*., 2013).

In conclusion, although blastocyst biopsy is the norm when performing PGS /PGD during IVF treatments; other alternatives, such as PB biopsy, should be taken into account in some specific situations, as in patients with poor ovarian response and a diagnosed female genetic abnormality.
